# Geranylgeranyl Pyrophosphate Promotes Profibrotic Factors and Collagen‐Specific Chaperone HSP47 in Fibroblasts

**DOI:** 10.1111/jcmm.70273

**Published:** 2024-12-23

**Authors:** Gracious R. Ross, Sanja Vodanovic‐Jankovic, Ivor J. Benjamin

**Affiliations:** ^1^ Cardiovascular Center Medical College of Wisconsin Milwaukee Wisconsin USA

**Keywords:** differentiation, fibroblasts, geranylgeranyl pyrophosphate, HSP47

## Abstract

Fibrosis, characterised by excessive extracellular matrix deposition, contributes to both organ failure and significant mortality worldwide. Whereas fibroblasts are activated into myofibroblasts, marked by phenotypic factors such as α‐smooth muscle actin (α‐SMA), periostin, fibroblast activation protein (FAP) and heat shock protein 47 (HSP47), the cellular processes of trans‐differentiation for fibrosis development remain poorly understood. Herein, we hypothesised that the molecular signalling of geranylgeranyl pyrophosphate (GGPP), a crucial biochemical molecule for protein prenylation, is essential in the regulation of profibrotic mechanisms for fibroblast‐to‐myofibroblast activation. To test this hypothesis, we demonstrated pharmacological inhibition of geranylgeranyl pyrophosphate synthase (GGPS1) significantly decreased TGF‐β1‐dependent myofibroblast differentiation assessed by reduced α‐SMA, periostin, FAP and HSP47 expression. Exogenous GGPP in the presence of GGPS1 inhibition restored TGF‐β1‐induced differentiation, supporting posttranslational requirements of GGPP modification during myofibroblast differentiation. Selective inhibition of either geranylgeranyl transferase or farnesyl transferase significantly impacted TGF‐β1‐induced myofibroblast α‐SMA and HSP47 expression. The importance of protein prenylation as a key regulator of myofibroblast differentiation was remarkably revealed by an unexpected decrease in HSP47 expression. In contrast, direct HSP47 inhibition not only suppressed TGF‐β1‐induced α‐SMA expression but surprisingly could not be rescued using exogenous GGPP. A selective role for the ER‐resident chaperone HSP47 expression downstream of GGPP was suggested when the effects of GGPS1 inhibition on periostin expression were counteracted by GGPP and geranylgeranyl transferase inhibition. Taken together, our findings underscore for the first time the functional role of cholesterol synthesis‐independent GGPP‐dependent pathway in fibroblast‐to‐myofibroblast transition and open new potential therapeutic targets for antifibrosis therapies.

## 1 Introduction

Fibrosis is an essential adaptive response after tissue injury but an excess accumulation of extracellular matrix (ECM) causes organ failure, which accounts for one‐third of all global deaths [1, 2]. Many reports have shown that the cellular processes of trans‐differentiation from fibroblasts to myofibroblasts characteristically involve profibrotic factors including α‐smooth muscle actin (α‐SMA) [3], periostin [4], fibroblast activation protein (FAP) [5] and the collagen‐specific molecular chaperone heat shock protein 47 (HSP47) [6]. A commonly used class of lipid‐lowering drugs, statins, which are inhibitors of 3‐hydroxy‐3‐methylglutaryl‐coenzyme A (HMG‐CoA)‐reductase, was recently reported to induce the de‐differentiation of activated myofibroblasts into fibroblasts [7]. This potential antifibrosis effect is mediated via the inhibitory effect on the cholesterol synthesis‐independent downstream pathway of HMGCoA reductase, which is considered to play a major role in the pleiotropic effects of statin therapy. An intermediary molecule in the cholesterol synthesis‐independent pathway, geranylgeranyl pyrophosphate (GGPP), an isoprenoid, appears to play a pivotal role in the myofibroblast de‐differentiation effect [7]. GGPP is synthesised by geranylgeranyl pyrophosphate synthase (GGPS1), which catalyses the trans‐addition of three molecules of isopentenyl pyrophosphate (IPP) onto dimethylallyl pyrophosphate (DMAPP) [8, 9]. GGPP is also known to be involved in the prenylation process—such as the addition of geranylgeranyl lipid groups by geranylgeranyl transferase (GGTase I or GGTase II) [8]—of various proteins involved in cell growth, differentiation and survival [10], specifically posttranslational modification of the Ras small GTPase superfamily of proteins (Rab, Rap and Rho families) [9]. Among profibrotic factors, the fibroblast HSP47 gene product of the serine proteinase H1 inhibitor family (SERPINH1) resides in the endoplasmic reticulum where it binds procollagen and facilitates collagen synthesis [6]. The essentiality of fibroblast HSP47 expression for postinjury recovery and repair was established recently by Molkentin and coworkers who demonstrated that fibroblast‐specific *HSP47* deficiency causes inadequate scar formation, ventricular rupture and death after acute myocardial infarction (AMI) in mice [11]. Recent human studies with implications from the profibrotic complications with COVID‐19 [12] to platelet‐mediated mechanisms of thromboembolic events [13] have heightened the interest in HSP47 as a potential therapeutic target [14, 15]. We considered that no prior studies have directly examined the specific role of GGPP in the underlying signalling mechanisms of HSP47 expression during the differentiation of fibroblasts into myofibroblasts. Hence, we examined the cholesterol synthesis‐independent signalling mechanisms of fibrogenesis during the trans‐activation of fibroblasts into myofibroblasts using biochemical and pharmacological tools in vitro. We tested the hypothesis that inhibition of GGPS1 arrests the trans‐differentiation of fibroblasts into myofibroblasts characterised by downregulation of profibrotic factors—α‐SMA, periostin, FAP and HSP47, a molecular chaperone—that promotes folding of collagen and precursor of fibrosis when de novo synthesis exceeds the degradation rate [16].

## 2 Materials and Methods

Human primary dermal fibroblasts (normal) [(ATCC PCS‐201‐010) and (ATCC PCS‐201‐012)] were purchased from ATCC (Manassas, VA) along with human primary cardiac fibroblasts (Cat. #: H‐6049) from Cell Biologics (Chicago, IL). GGPP and Mevalonate were purchased from Cayman Chemical (Ann Arbor, MI), while recombinant human TGF‐β1 was purchased from Peprotech (Cranbury, NJ), and rhosin hydrochloride, gallein and GGTI 298 were purchased from Tocris/Bio‐Techne Corporation (Minneapolis, MN). Digeranyl bisphosphonate, HY‐124817 and FTI‐277 hydrochloride solution were purchased from MedChemExpress LLC (Monmouth Junction, NJ). CellTiter‐Glo 2.0 Cell Viability Assay (Cat # G9241) was purchased from Promega (Madison, WI). Antibodies to periostin (PA5‐34641), PGGT1B (H00005229‐MO2), were obtained from Thermo Fisher (Waltham, MA), anti‐α‐SMA antibody (ab7817) from Abcam (Waltham, MA), HSP47 (sc‐5293) and GGPS1 (sc‐271679) from Santa Cruz (Dallas, TX), α/β‐tubulin (2148S) and GAPDH (97166S) antibodies from Cell Signalling (Beverly, MA) and FAP alpha/FAP antibody (NBP2‐66844) from Novusbio (Centennial CO). HRP (horseradish peroxidase)‐conjugated goat anti‐rabbit IgG (H + L) and donkey anti‐mouse IgG (H + L) highly cross‐adsorbed secondary antibodies were also purchased from Thermo Fisher.

### 2.1 Method for Fibroblast Differentiation Into Activated Myofibroblasts

Different human fibroblasts were plated at 6000 cells/cm^2^ with DMEM media (10% FBS) and incubated at 37°C under 5% CO_2_. Following 24 h, fibroblasts were either treated with TGF‐β1 (5 ng/mL) or kept as control in DMEM media (2.5% FBS). After 48–72 h, the fibroblasts/myofibroblasts were rinsed with Dulbecco's PBS and assayed for myofibroblast markers like α‐SMA, FAP, periostin and HSP47.

### 2.2 Protocol for GGPP‐Dependent Signalling Mechanisms in Fibroblast–Myofibroblast Trans‐Differentiation

Following the above protocol for differentiation of fibroblasts into myofibroblasts, either inhibitors or activators of specific enzymatic steps were added along with TGF‐β1 and incubated for 72 h for assays of various enzymatic reactions. The targeted signalling steps and corresponding modulators were used to probe geranylgeranyl diphosphate synthase (GGPS1) inhibition by DGBP (10 μM), geranylgeranyl transferase I (GGTase I) inhibitor (GGTI‐298, 5 μM), FTI‐277 hydrochloride (5 μM), an inhibitor of farnesyl transferase (FTase), rhosin hydrochloride (10 μM), a Rho GTPase inhibitor, ROCK inhibition by Y27632 (10 μM), gallein (10 μM), an inhibitor of G protein βγ subunit‐dependent signalling, HSP47 inhibition by HY124817 (10 μM) and the product of GGPS1 enzymatic reaction, GGPP (25 μM).

### 2.3 Protein Isolation and Immunoblotting

When cells were ready for assay, RIPA lysis buffer was used to dissociate and lyse the cells for protein isolation as per manufacturer protocol. Protein concentrations of samples were estimated using Pierce BCA protein assay kit (Cat # 23225). Standard western protocols were followed [17] with respective primary antibodies (dilutions: α‐SMA, 1:500, HSP47, 1:500, periostin, 1:500, FAP, 1:500, GGPS1, 1:500, PGGT1B, 1:500, β‐tubulin, 1:500 and GAPDH, 1:1000) and HRP‐conjugated secondary antibodies (1:4000). All samples were immunoblotted simultaneously and repeated at least twice. Respective immunoblots of proteins of interest and housekeeping proteins including control samples were normalised and quantified using Image J software.

### 2.4 Statistical Analysis

Continuous variables were analysed by two‐sample *t*‐test or one‐way analysis of variance (Brown–Forsythe) followed by Dunnett's T3 multiple‐comparisons test. *p* < 0.05 was considered significant.

## 3 Results

### 3.1 GGPS1 Inhibition Decreases TGF‐β1‐Induced Fibroblast–Myofibroblast Trans‐Differentiation

**FIGURE 1:**
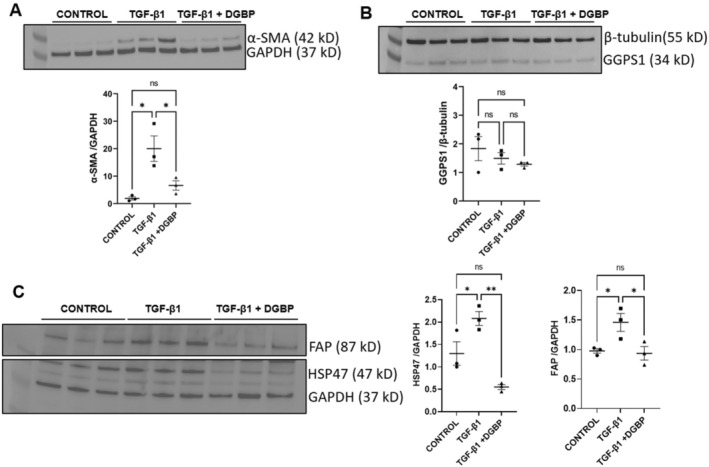
GGPS1 inhibition decreases TGF‐β1‐induced fibroblast–myofibroblast trans differentiation. (A) Immunoblot showing incubation of TGF‐β1 (5 ng/mL) for 72 h increased the expression of α‐SMA without any significant difference in the GAPDH expression while inhibition of GGPS1 by co‐administration of DGBP (10 μM) significantly decreased the TGF‐β1‐induced α‐SMA expression. Lower panel displays the bar graph representing the mean values of corresponding band intensities of the above blot. (B) Immunoblot showing incubation of TGF‐β1 (5 ng/mL) either in the presence or absence of DGBP (10 μM) for 72 h did not affect the expression of either geranylgeranyl pyrophosphate synthase 1 (GGPS1) enzyme or β‐tubulin. Lower panel displays the bar graph representing the mean values of corresponding band intensities of the above blot. (C) Immunoblot showing expression of FAP, HSP47 and GAPDH either in the presence of TGF‐β1‐ or co‐administration of DGBP. Right side bar graph depicts the respective mean values of band intensities. DGBP has significantly (*p* < 0.05) decreased the TGF‐β1‐induced increase in the expression of FAP. Similarly, the expression of HSP47 was also significantly (*p* < 0.01) decreased by DGBP in the TGF‐β1 group. One way ANOVA, *N* = 3; *p < 0.05 and **p < 0.01 considered significant.

In our recently published studies of human hearts with chronic heart failure, we tested the hypothesis that the effects of statin therapy could promote tissue profibrotic remodelling through the cholesterol synthesis‐independent pathway involving the GGPP in vivo [7]. Indeed, we observed that myofibroblasts of patients receiving statin therapy exhibited significantly lower α‐SMA expression, a biomarker of cellular differentiation, compared with cellular phenotypes in individuals without statin treatment [7]. TGF‐β1 (5 ng/mL) is widely used to induce differentiation of fibroblasts into activated myofibroblasts [18, 19]. As shown in Figure 1A, the incubation of fibroblasts with 10 μM digeranyl bisphosphate, an inhibitor of GGPS1 [7, 20], significantly (*p* < 0.05) inhibited the TGF‐β1‐induced myofibroblast differentiation in parallel with significantly decreased α‐SMA expression compared with the TGF‐β1 treatment alone. The expression of GGPS1 enzyme was appreciably unchanged after TGF‐β1 treatment either in the presence or absence of DGBP (Figure 1B). In parallel, DGBP treatment significantly (*p* < 0.05) decreased the TGF‐β1‐induced increase in the expression of FAP (Figure 1C), establishing complementary molecular evidence on the effects of GGPS1 inhibition on myofibroblast activation. Because collagen‐specific molecular chaperone HSP47 was also significantly (*p* < 0.01) decreased by DGBP in the TGF‐β1 treatment group (Figure 1C), such evidence validates our bona fide system for testing a critical role involving GGPS signalling in fibrogenesis and procollagen processing [11, 21] in vitro.

### 3.2 Role of Cholesterol Synthesis‐Independent Pathway in Fibroblast–Myofibroblast Trans‐Differentiation

**FIGURE 2:**
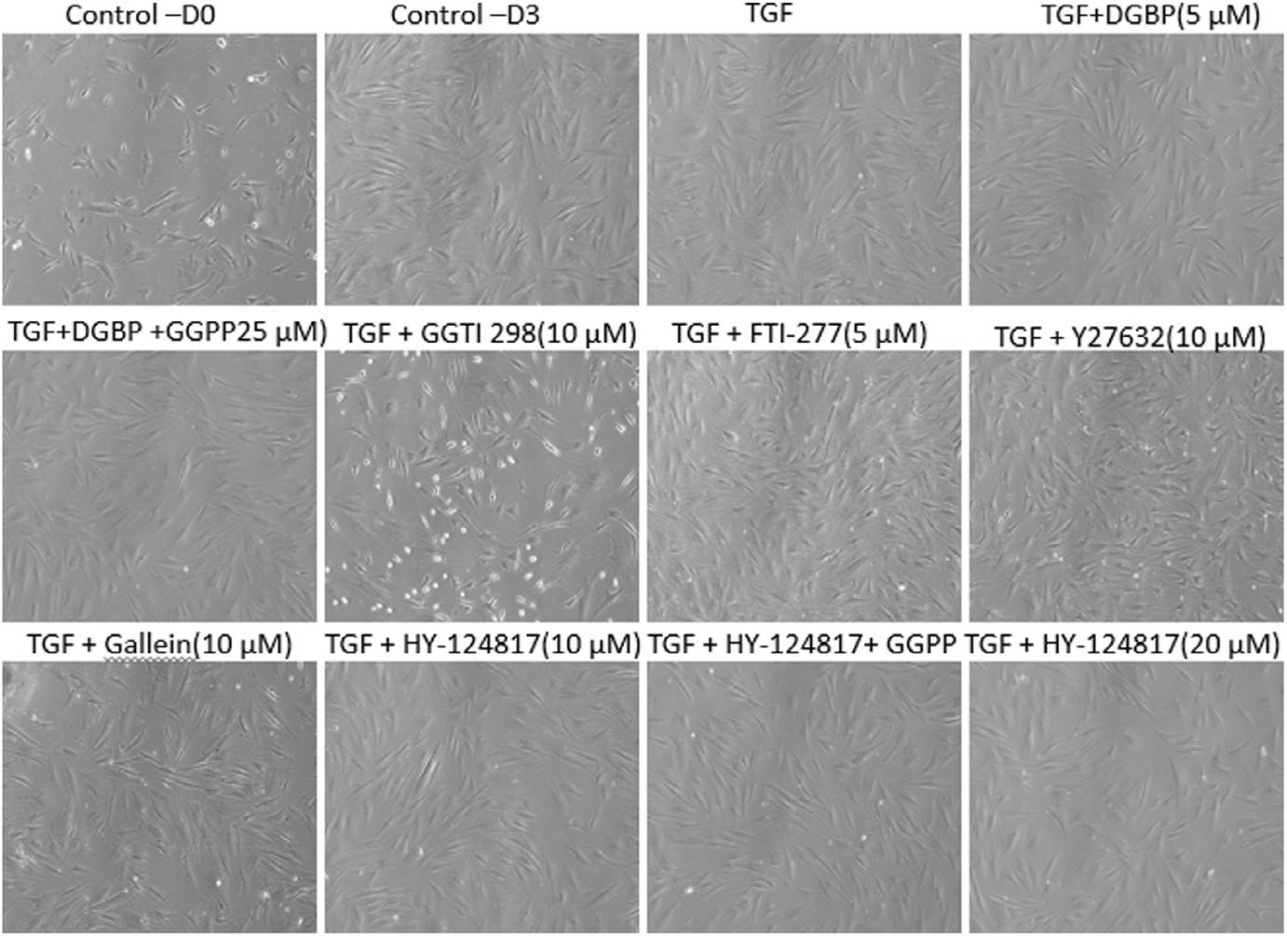
Cell morphology and viability under various treatment conditions. Representative bright field images (10×) of fibroblasts/myofibroblasts showing viability of the cells at various conditions following treatments for 72 h with TGF‐β1 (5 ng/mL), inhibition of GGPS1 by DGBP (10 μM), co‐administration of GGPP (25 μM), inhibition of geranylgeranyl transferase by GGTI‐298 (10 μM), inhibition of farnesyl transferase by FTI‐277 (5 μM), inhibition of Rho GTPase by rhosin (10 μM), inhibition of Rho‐associated kinase (ROCK) by Y27632 (10 μM), inhibition of G protein βγ subunit‐dependent signalling by gallein (10 μM), direct inhibition of HSP47 by HY124817 (10 μM) or co‐administration with GGP (25 μM). D0: Day 0; D3: Day 3, following plating of cells.

**FIGURE 3:**
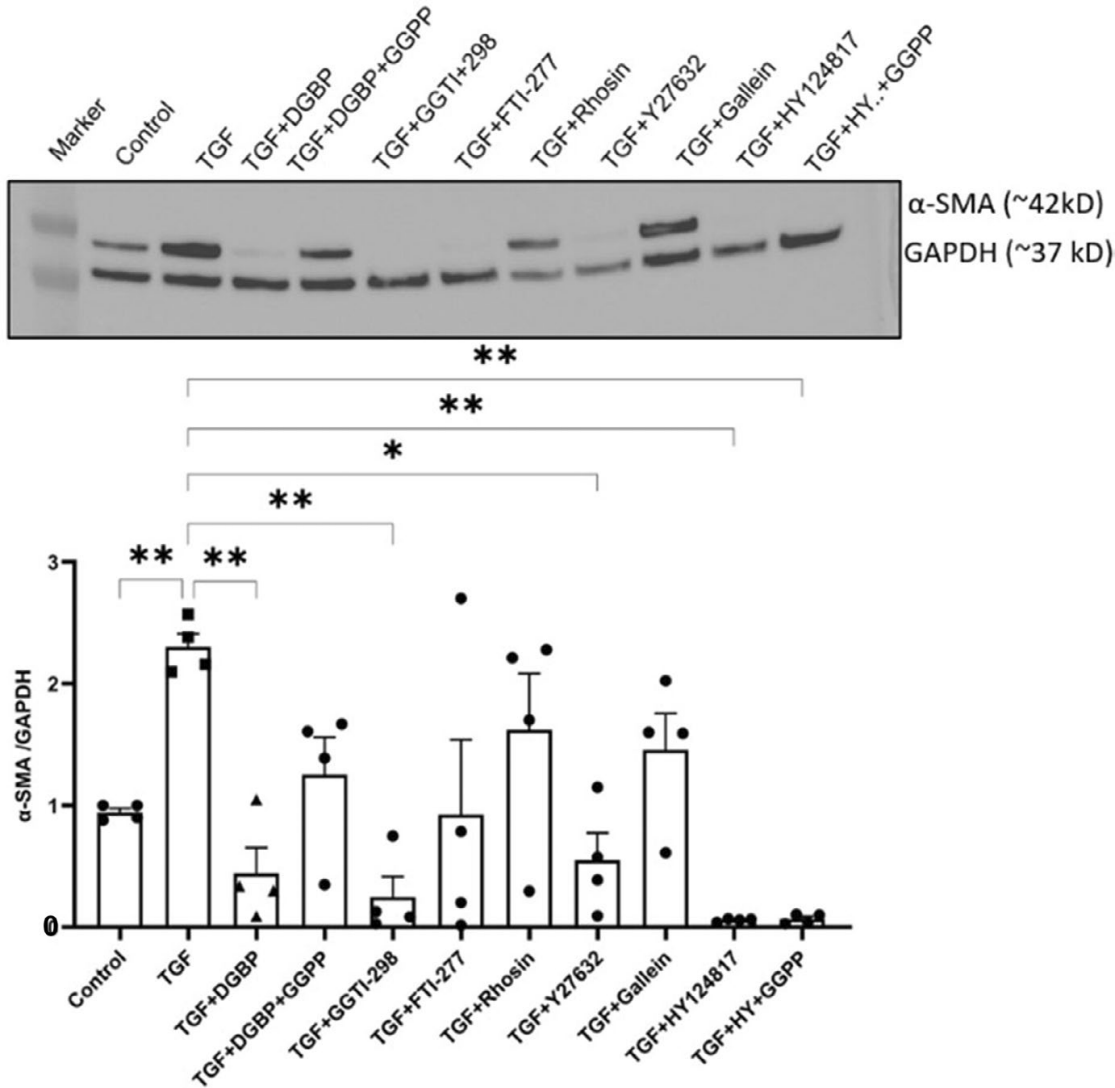
Cholesterol synthesis‐independent pathway mechanisms involved in fibroblast–myofibroblast trans‐differentiation. Top panel shows the representative immunoblot of all treatment conditions and bottom panel depicts the bar graph with respective mean values of band intensities. Exogenous administration of GGPP (25 μM) prevented the DGBP (10 μM)—induced inhibition of α‐SMA, a marker of activated myofibroblasts. In the presence of GGPP (TGF + DGBP + GGPP), there was no significant difference in the α‐SMA expression compared to TGF group. Inhibition of GGT by GGTI‐298 (5 μg/mL) significantly decreased the TGF‐induced α‐SMA expression, and inhibition of farnesyl transferase by FTI‐277 (5 μM) did not significantly affect the TGF‐β1‐induced α‐SMA expression. Similarly, either inhibition of Rho GTPase by rhosin (10 μM) or inhibition of G protein βγ subunit‐dependent signalling by gallein (10 μM) did not significantly affect the TGF‐β1‐induced increased α‐SMA expression. However, inhibition of Rho‐associated kinase (ROCK) by Y27632 (10 μM) significantly decreased the TGF‐β1‐induced α‐SMA expression; and direct inhibition of HSP47 by HY124817 (10 μM) significantly inhibited the TGF‐β1‐induced α‐SMA expression that was not sensitive to exogenous administration of GGPP (25 μM) of mevalonate (300 μM). One‐way ANOVA, *N* = 3; *p < 0.05 or **p < 0.01 considered significant.

First, we set out to directly determine whether either the GGPP‐dependent and/or the cholesterol synthesis‐independent pathways are similarly involved in the signalling mechanisms of profibrotic HSP47^+^ myofibroblast differentiation. We first established that cellular viability of fibroblasts/myofibroblasts was unaffected in all the treatment groups using specific pharmacological inhibitors and agonists during the experimental conditions over 72 h (Figure 2). In addition, we performed the viability assay using CellTiter Glo in all the groups. Compared to the control groups, viability changes were observed across various groups with the maximum change observed in TGF + DGBP + GGPP (78% ± 1.3% viable) and TGF + gallein (71% ± 1.7%) groups (Figure S2). However, the overall cellular viability suggests that there was no major cytotoxicity in any group. The inhibitory effects of DGBP—induced in myofibroblast activation, using profibrotic biomarker α‐SMA expression—were entirely reversed by exogenous administration of the product of GGPS1 enzyme, GGPP (25 μM) (Figure 3). There was no significant (*p* = 0.48) difference in the α‐SMA expression between treatment group in the presence of GGPP (TGF + DGBP + GGPP) compared with TGF‐β1 group. However, the statistical difference between the control group and the TGF + DGBP + GGPP group did not reach significance (*p* = 0.16) due to one outlier type value (0.35, 1.60, 1.67 and 1.39), but trending towards significance. Geranylation of downstream target proteins by GGPP is catalysed by geranylgeranyl transferase (GGT) [8]. Furthermore, inhibition of GGT by GGTI‐298 (5 μg/mL), which significantly (*p* < 0.05) decreases TGF‐ β1‐induced α‐SMA expression, mimics the effects of GGPS inhibition by DGBP. Thus, geranylation of downstream signalling molecules is involved in the myofibroblast differentiation, whereas farnesylation is not. Indeed, the TGF‐β1‐induced α‐SMA expression is unaffected by inhibition of farnesyl transferase by FTI‐277 (5 μM) (*p* = 0.77). Similarly, neither inhibition of Rho GTPase by rhosin (10 μM) nor inhibition of G protein βγ subunit‐dependent signalling by gallein (10 μM) significantly affected the TGF‐ β1‐induced increased α‐SMA expression (Figure 3). However, inhibition of Rho‐associated kinase (ROCK) by Y27632 (10 μM) significantly decreased the TGF‐β1‐induced α‐SMA expression. Of special note, the direct inhibition of HSP47 by HY124817 (10 μM) associated with significantly (*p* < 0.05) decreased TGF‐β1‐induced α‐SMA expression was independent of GGPP (25 μM) with exogenous administration (*p* < 0.05) (Figure 3).

### 3.3 Signalling Mechanisms Involved in the TGF‐β1‐Induced Upregulation of HSP47 Expression

**FIGURE 4:**
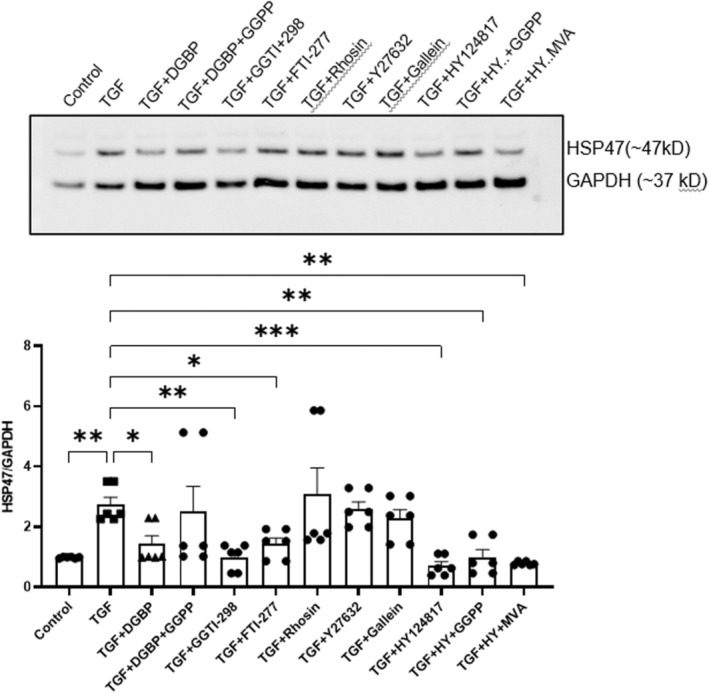
Signalling mechanisms involved in the TGF‐β1‐induced upregulation of HSP47. Top panel shows the representative immunoblot of all treatment conditions and bottom panel depicts the bar graph with respective mean values of band intensities. Exogenous administration of GGPP (25 μM) prevented the DGBP (10 μM)—induced inhibition of HSP47. In the presence of GGPP (TGF + DGBP + GGPP), there was no significant difference in the HSP47 expression compared to TGF group. Inhibition of GGT by GGTI‐298 (5 μg/mL) significantly decreased the TGF‐β1‐induced HSP47 expression‐like inhibition of farnesyl transferase by FTI‐277 (5 μM). However, inhibition of Rho GTPase by rhosin (10 μM), inhibition of Rho‐associated kinase (ROCK) by Y27632 (10 μM) or inhibition of G protein βγ subunit‐dependent signalling by Gallein (10 μM) did not significantly affect the TGF‐β1‐induced increased HSP47 expression. Direct inhibition of HSP47 by HY124817 (10 μM) significantly inhibited the TGF‐β1‐induced HSP47 expression, which was not sensitive to exogenous administration of GGPP (25 μM) of mevalonate (300 μM). One way ANOVA, *N* = 6; *p < 0.05, **p < 0.01 and ***p < 0.001 considered significant.

The surprising effects of the DGBP, an inhibitor of GGPS, on decreased levels in the HSP47 expression (Figure 1C) led us to hypothesise that cholesterol synthesis‐independent signalling involving the GGPP‐dependent pathway regulates myofibroblast‐specific HSP47 expression. To test this hypothesis through pharmacological modulation in fibroblasts, the addition of GGPP (25 μM) countered DGBP‐induced suppression of TGF‐β1‐induced HSP47 expression, underscoring GGPP's pivotal role in HSP47 regulation during fibroblast trans‐differentiation (Figure 4). GGPP could counteract and neutralise DGBP effects (TGF + DGBP + GGPP) on HSP47 expression (*p* = 0.99) compared to TGF‐β1 group. Similar to GGPS, the inhibition of GGT by GGTI‐298 (5 μg/mL) significantly (*p* < 0.01) reduced TGF‐β1‐induced HSP47 expression, suggesting downstream signalling molecules' geranylation modulates HSP47 during fibroblast–myofibroblast transition. Inhibition of farnesyl transferase, by FTI‐277 (5 μM), also significantly (*p* < 0.05) affected TGF‐β1‐induced HSP47 expression, indicating potential role of farnesylation in HSP47 expression as well. Conversely, Rho GTPase inhibition, ROCK inhibition and G protein βγ subunit‐dependent signalling inhibition showed no significant effect on TGF‐β1‐induced HSP47 expression (Figure 4). HSP47 inhibitor HY124817 (10 μM) significantly reduced TGF‐β1‐induced HSP47 expression, even in the presence of either GGPP (25 μM) or mevalonate (300 μM) (*p* < 0.05).

### 3.4 Signalling Mechanisms Regulating TGF‐β1 Induced Periostin Expression

**FIGURE 5:**
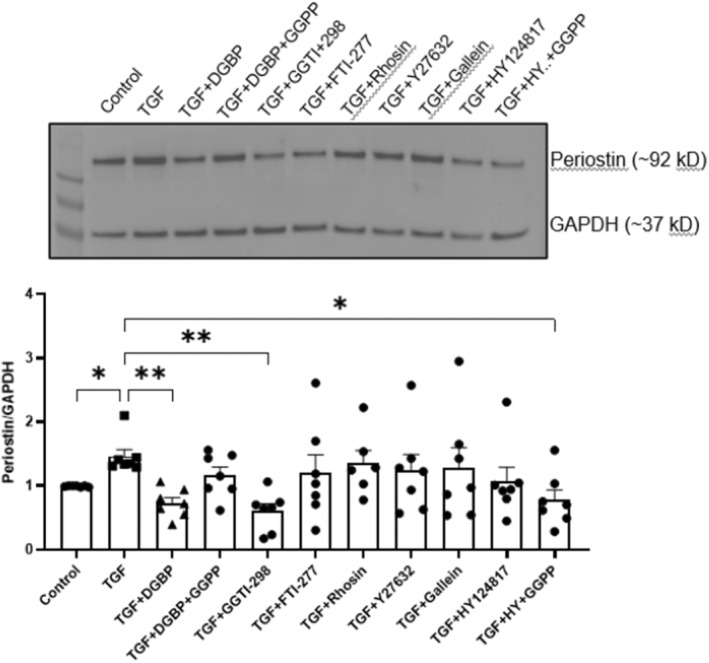
Signalling mechanisms regulating TGF‐β1‐induced periostin expression. Top panel shows the representative immunoblot of all treatment conditions and bottom panel depicts the bar graph with respective mean values of band intensities. Exogenous administration of GGPP (25 μM) prevented the DGBP (10 μM)—induced inhibition of periostin. In the presence of GGPP (TGF + DGBP + GGPP), there was no significant difference in the periostin expression compared to TGF group. Inhibition of GGT by GGTI‐298 (5 μg/mL) significantly decreased the TGF‐β1‐induced periostin expression unlike inhibition of farnesyl transferase by FTI‐277 (5 μM). However, inhibition of Rho GTPase by rhosin (10 μM), inhibition of Rho‐associated kinase (ROCK) by Y27632 (10 μM) or inhibition of G protein βγ subunit‐dependent signalling by gallein (10 μM) did not significantly affect the TGF‐β1‐induced increased periostin expression. Direct inhibition of HSP47 by HY124817 (10 μM) did not significantly affect the TGF‐β1‐induced periostin expression, which was not sensitive to exogenous administration of GGPP (25 μM) of mevalonate (300 μM). One way ANOVA, *N* = 6; *p < 0.05 and **p < 0.01 considered significant.

As activated myofibroblasts are key cellular mediators of tissue fibrosis, and periostin has been shown to play an important role in regulating myofibroblast function, we next examined the effects of GGPS1 inhibition on periostin expression during the fibroblasts–myofibroblast trans‐differentiation. As shown in Figure 5, immunoblotting of fibroblasts/myofibroblast lysates showed significant (*p* < 0.05) increase in periostin expression following TGF‐β1 treatment while GGPS1 inhibition by DGBP significantly (*p* < 0.01) decreased this TGF‐β1‐induced increase. Co‐incubation with GGPP similarly reversed the effects of DGBP (*p* = 0.58). In addition, inhibition of GGT by GGTI‐298 also significantly decreased the TGF‐β1‐induced increase in periostin expression. However, inhibition of farnesyl transferase by FTI‐277, inhibition of Rho GTPase by Rhosin, inhibition of ROCK by Y27632 or inhibition of G protein βγ subunit‐dependent signalling by gallein did not significantly affect the TGF‐β1‐induced increase in periostin expression (Figure 5).

### 3.5 Expression of Protein Geranylgeranyl Transferase Type I, Beta Subunit (PGGT1B) During Fibroblast/Myofibroblast Trans‐Differentiation

PGGT1B catalyses the transfer of a geranyl‐geranyl moiety from geranyl‐geranyl pyrophosphate (GGPP) to both cysteines in Rab proteins with an ‐XXCC, ‐XCXC and ‐CCXX C‐terminal, such as RAB1A, RAB3A and RAB5A respectively [22]. Therefore, we determined if there is any significant change in the expression of PGGT1B during the TGF‐β1‐induced differentiation of fibroblasts into myofibroblasts, and whether affected by increased levels of GGPP, inhibition of GGPS1, inhibition of farnesyl transferase, inhibition of Rho GTPase, inhibition of ROCK or inhibition of G protein βγ subunit‐dependent signalling. As displayed in Figure S1, there were no significant differences in the expression of PGGT1B across all treatment conditions.

## 4 Discussion

The salient findings from this study are as follows: (1) Inhibiting GGPS1 significantly reduced fibroblast‐to‐myofibroblast differentiation and expression of α‐SMA, periostin, FAP and HSP47; (2) exogenous GGPP, the product of GGPS1, preserved the TGF‐β1‐induced myofibroblast differentiation during GGPS1 inhibition; (3) inhibition of GGT1 also significantly decreased the TGF‐β1‐induced activation of fibroblasts into myofibroblasts; (4) neither GGPS1 nor GGT1 expression changed during differentiation; (5) direct HSP47 inhibition significantly inhibited TGF‐β1‐induced fibroblast‐to‐myofibroblast differentiation; and (6) exogenous GGPP did not rescue the direct HSP47 inhibition‐induced decrease in differentiation.

Fibroblasts, a type of mesenchymal cell, are essential for wound healing and tissue repair. When tissues are injured or inflamed, fibroblasts transform into myofibroblasts, specialised contractile cells that contribute to extracellular matrix (ECM) deposition and remodelling during tissue repair [23]. This differentiation process is orchestrated by a range of cytokines, growth factors and ECM proteins, leading to changes in gene expression, morphology and function. Numerous studies, both in vitro and in vivo, have delved into the intricate molecular mechanisms governing fibroblast‐to‐myofibroblast differentiation [24, 25, 26]. In addition, several transcription factors also regulate this process [27]. Recent research highlights the intriguing potential of statins, commonly used lipid‐lowering drugs that inhibit HMG‐CoA reductase, to prompt myofibroblast de‐differentiation into fibroblasts [7]. This antifibrotic effect is achieved via the inhibitory effect on the cholesterol synthesis‐independent downstream pathway of HMGCoA reductase that mediates the pleiotropic effects of statins [28]. In this context, GGPP, an isoprenoid, plays a pivotal role in the myofibroblast de‐differentiation [7]. GGPP is involved in protein prenylation process—such as addition of geranylgeranyl lipid groups to proteins involved in cell growth, differentiation and survival [10], by geranylgeranyl transferase (GGTase I or GGTase II) [8], specifically posttranslational modification of the Ras small GTPase superfamily of proteins [9]. Myofibroblast transition frequently involves profibrotic factors like α‐SMA [3], HSP47 [11], periostin [4], FAP [5] and collagen [29]. However, the precise role of GGPP and its underlying signalling mechanisms in fibroblast‐to‐myofibroblast differentiation remain unclear. Hence, this study aimed to uncover how GGPP signalling influences the profibrotic traits during fibroblast‐to‐myofibroblast transition. The hypothesis tested was that suppressing GGPS1 during fibroblast‐to‐myofibroblast transition could downregulate profibrotic factors like α‐SMA, periostin, FAP and HSP47—a molecular chaperone crucial for collagen folding, which can lead to fibrosis when its synthesis surpasses degradation [16]. Inhibition of GGPS1 by DGBP [7, 20] significantly hindered TGF‐β1‐induced myofibroblast differentiation. This inhibition was evident from reduced α‐SMA expression, with no change in GGPS1 expression by either TGF‐β1 or DGBP. Furthermore, DGBP lowered TGF‐β1‐induced FAP expression, corroborating the impact of GGPS1 inhibition on myofibroblast activation. Similarly, HSP47, a stress‐inducible collagen‐specific molecular chaperone in procollagen processing [11, 21], was also significantly decreased by DGBP in the TGF‐β1 group. This suggests GGPS's critical role in regulating collagen processing via HSP47. Together, GGPP levels emerge as vital for myofibroblast activation and regulation of HSP47's downstream collagen processing. As a note, while we did not directly assess GGPP levels to ensure the efficacy of DGBP due to technical challenges, the observed reversal of the effect when external GGPP, the product of GGPS1 was added, strongly supports the effective inhibition of GGPP production by DGBP, with additional evidence from other reports {Wiemer, 2007 #62}. The study delved into the mechanisms of the cholesterol synthesis‐independent pathway, focusing on GGPP dependence and subsequent signalling using specific pharmacological inhibitors and agonists. Exogenously administered GGPP (25 μM), derived from the GGPS1 enzyme, countered DGBP‐induced inhibition of TGF‐β1‐induced fibroblast‐to‐myofibroblast differentiation. GGPP is typically used by GGT for geranylation of downstream proteins [30] and inhibiting GGT (using GGTI‐298) significantly reduced TGF‐β1‐induced α‐SMA expression, implying geranylation of downstream signalling molecules is involved in myofibroblast differentiation. Notably, inhibiting farnesyl transferase via FTI‐277 did not significantly affect TGF‐β1‐induced α‐SMA expression, indicating a potential difference between geranylation and farnesylation in this context. Directly inhibiting HSP47 effectively suppressed TGF‐β1‐induced α‐SMA expression. Notably, this inhibition was not reversed by exogenous GGPP (25 μM) or mevalonate (300 μM). This indicates that HSP47 is a key player in fibroblast‐to‐myofibroblast differentiation, regulated downstream of GGPP in the cholesterol synthesis‐independent pathway of myofibroblast activation. This novel observation led to investigation of HSP47 expression regulation by the cholesterol synthesis‐independent pathway and GGPP dependence, revealing GGPP's critical role.

Exogenously applied GGPP mitigated DGBP‐induced suppression of TGF‐β1‐induced HSP47 expression. This confirmed the significance of GGPP, the product of GGPS1, in regulating HSP47 expression during fibroblast‐to‐myofibroblast transition. Inhibiting GGT with GGTI‐298 significantly decreased TGF‐β1‐induced HSP47 expression, resembling the effects of GGPS inhibition via DGBP. This supports the role of geranylation in regulating HSP47 expression during fibroblast‐to‐myofibroblast differentiation. Noticeably, inhibition of farnesyl transferase did not affect α‐SMA expression but it did decrease HSP47 expression that is considered upstream of α‐SMA and upregulates it. Other compensatory mechanisms or parallel pathways may maintain α‐SMA expression even in the face of decreased HSP47 levels. Further research is warranted to fully elucidate the interplay between these factors in the context of myofibroblast differentiation. Conversely, inhibiting other pathways did not notably affect TGF‐β1‐induced HSP47 expression. Surprisingly, direct inhibition of HSP47 did not significantly impact periostin expression. Periostin, crucial for cell adhesion, migration, proliferation and ECM remodelling [31], was influenced by GGPS1 inhibition. Immunoblotting revealed a notable increase in periostin expression post‐TGF‐β1 treatment, which DGBP significantly reduced. This effect was counteracted by GGPP co‐incubation. Inhibiting GGT also reduced TGF‐β1‐induced periostin expression. However, inhibiting farnesyl transferase, Rho GTPase, ROCK or G protein βγ subunit‐dependent signalling had limited impact. It has to be noted that inhibition of HSP47 results in a drastic decrease in its expression as opposed to GGPS1 inhibition. The mechanism behind the significant decrease in HSP47 expression warrants further investigation. While our current study did not specifically investigate mRNA stability, posttranslational regulation, protein stability or turnover of HSP47, we hypothesise that the observed changes could involve such mechanisms that could be explored in future studies.

**FIGURE 6:**
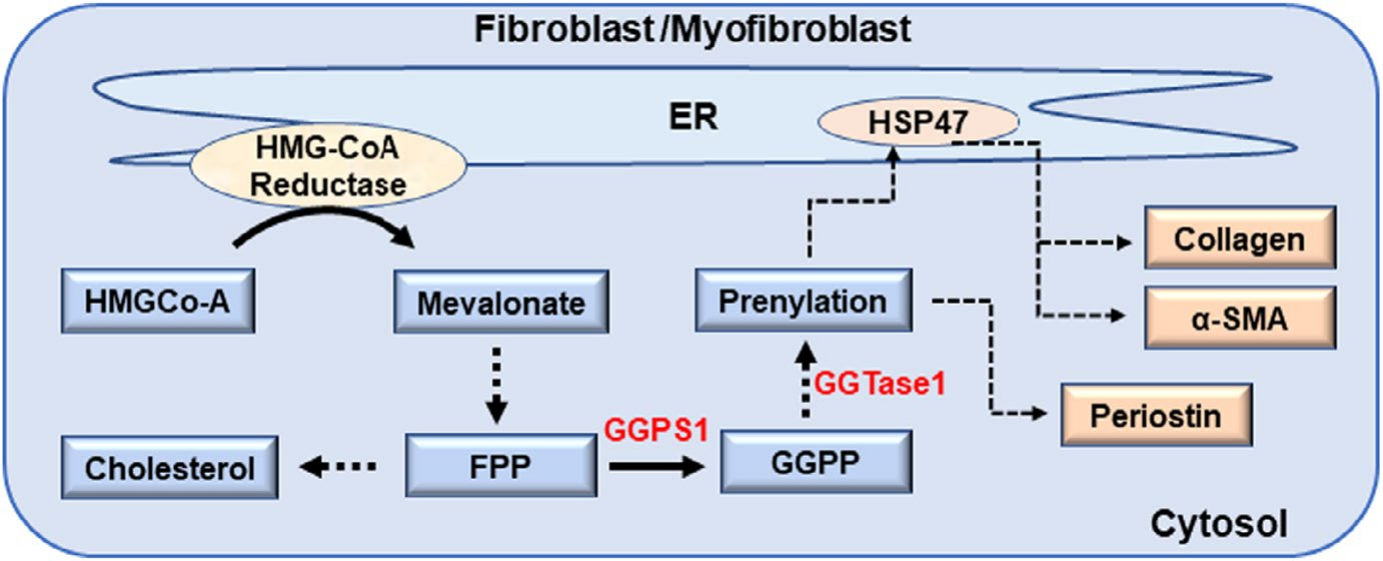
Schema: GGPP signalling in fibroblast/myofibroblast differentiation. In fibroblasts, 3‐hydroxy‐3‐methylglutaryl‐coenzyme A (HMG‐CoA) reductase located in the ER converts HMG‐CoA into mevalonate that is converted by series of enzymatic steps into farnesyl pyrophosphate (FPP). Geranylgeranyl pyrophosphate is synthesised by GGPS1 (geranylgeranyl disphosphate synthase (1) from FPP. Geranylgeranyl transferase (GGTase 1) catalyses the prenylation of various proteins. Through subsequent potential signalling steps, profibrotic process including collagen processing and α‐SMA (α‐smooth muscle actin) expression happens with the involvement of HSP47, while periostin expression occurs independent of HSP47. Solid arrows: Direct step; Broken arrows: Multiple steps involved.

In conclusion, as depicted in the graphical depiction (Figure 6), the downstream cholesterol synthesis‐independent signalling pathway of HMG‐CoA reductase, regulated by GGPP and protein prenylation, orchestrates the fibroblast‐to‐myofibroblast transition. This is underscored by significant changes in profibrotic factors like α‐SMA, periostin and HSP47. Further research is essential to grasp the intricate molecular interactions and mechanisms guiding myofibroblast differentiation and to uncover the therapeutic potential of targeting these cholesterol synthesis‐independent pathway molecules in fibrotic diseases.

## Author Contributions

**Gracious R. Ross:** conceptualization (equal), data curation (lead), formal analysis (equal), methodology (lead), project administration (equal), software (lead), visualization (equal), writing – original draft (equal). **Sanja Vodanovic‐Jankovic:** methodology (supporting), visualization (equal). **Ivor J. Benjamin:** conceptualization (equal), data curation (supporting), formal analysis (supporting), funding acquisition (lead), investigation (lead), methodology (supporting), project administration (supporting), resources (lead), software (supporting), supervision (lead), validation (lead), visualization (supporting), writing – original draft (supporting), writing – review and editing (lead).

## Conflicts of Interest

The authors declare no conflicts of interest.

## Supporting information


**Figure S1.** Expression of protein geranylgeranyl transferase type I, beta subunit (PGGT1B) during fibroblast/myofibroblast trans‐differentiation. Top panel shows the representative immunoblot of all treatment conditions and bottom panel depicts the bar graph with respective mean values of band intensities. TGF‐β1, increased levels of GGPP, inhibition of GGPS1, inhibition of farnesyl transferase, inhibition of Rho GTPase, inhibition of Rho‐associated kinase (ROCK) or inhibition of G protein βγ subunit‐dependent signalling did not significantly affect the expression of PGGT1B across all treatment conditions. One‐way ANOVA, *N* = 3; *p < 0.05 considered significant.
**Figure S2.** CellTiter Glo luminescent viability assay. Compared to the control groups, the viability changes across different groups with significance included 86% ± 3% (TGF), 78% ± 2% (TGF + DGBP + GGPP), 88% ± 2% (TGF + GGTI‐298), 85% ± 2% (TGF + FTI‐277), 71% ± 1.3% (TGF + gallein) and 90% ± 3% (TGF + HY124817 + GGPP). However, the overall cellular viability suggests that there was no major cytotoxicity in any group. One‐way ANOVA; *N* = 3; *p < 0.05 considered significant.

## Data Availability

The data that support the findings of this study are openly available in figshare at https://doi.org/10.6084/m9.figshare.24011106.v1.

## References

[1] D. C. Rockey , P. D. Bell , and J. A. Hill , “Fibrosis—A Common Pathway to Organ Injury and Failure,” New England Journal of Medicine 372, no. 12 (2015): 1138–1149, 10.1056/NEJMra1300575. 25785971

[2] R. A. F. Clark , “To Scar or Not to Scar,” New England Journal of Medicine 385, no. 5 (2021): 469–471, 10.1056/NEJMcibr2107204. 34320296

[3] B. Hinz , G. Celetta , J. J. Tomasek , G. Gabbiani , and C. Chaponnier , “Alpha‐Smooth Muscle Actin Expression Upregulates Fibroblast Contractile Activity,” Molecular Biology of the Cell 12, no. 9 (2001): 2730–2741, 10.1091/mbc.12.9.2730. 11553712 PMC59708

[4] C. G. Elliott , J. Wang , X. Guo , et al., “Periostin Modulates Myofibroblast Differentiation During Full‐Thickness Cutaneous Wound Repair,” Journal of Cell Science 125, no. Pt 1 (2012): 121–132, 10.1242/jcs.087841. 22266908 PMC3269025

[5] A. A. Fitzgerald and L. M. Weiner , “The Role of Fibroblast Activation Protein in Health and Malignancy,” Cancer Metastasis Reviews 39, no. 3 (2020): 783–803, 10.1007/s10555-020-09909-3. 32601975 PMC7487063

[6] K. Nagata , “HSP47 as a Collagen‐Specific Molecular Chaperone: Function and Expression in Normal Mouse Development,” Seminars in Cell & Developmental Biology 14, no. 5 (2003): 275–282, 10.1016/j.semcdb.2003.09.020. 14986857

[7] L. Emelyanova , A. Sra , E. G. Schmuck , et al., “Impact of Statins on Cellular Respiration and De‐Differentiation of Myofibroblasts in Human Failing Hearts,” ESC Heart Failure 6, no. 5 (2019): 1027–1040, 10.1002/ehf2.12509. 31520523 PMC6816080

[8] A. R. Foley , Y. Zou , J. E. Dunford , et al., “GGPS1 Mutations Cause Muscular Dystrophy/Hearing Loss/Ovarian Insufficiency Syndrome,” Annals of Neurology 88, no. 2 (2020): 332–347, 10.1002/ana.25772. 32403198 PMC7496979

[9] S. L. Haney , V. S. Wills , D. F. Wiemer , and S. A. Holstein , “Recent Advances in the Development of Mammalian Geranylgeranyl Diphosphate Synthase Inhibitors,” Molecules 22, no. 6 (2017): 886, 10.3390/molecules22060886. 28555000 PMC5902023

[10] W. Su , N. M. Chapman , J. Wei , et al., “Protein Prenylation Drives Discrete Signaling Programs for the Differentiation and Maintenance of Effector T(reg) Cells,” Cell Metabolism 32, no. 6 (2020): 996–1011.e7, 10.1016/j.cmet.2020.10.022. 33207246 PMC7887758

[11] H. Khalil , O. Kanisicak , R. J. Vagnozzi , et al., “Cell‐Specific Ablation of Hsp47 Defines the Collagen‐Producing Cells in the Injured Heart,” JCI Insight 4, no. 15 (2019): e128722, 10.1172/jci.insight.128722. 31393098 PMC6693833

[12] A. Puzyrenko , E. R. Jacobs , N. Padilla , et al., “Collagen‐Specific HSP47(+) Myofibroblasts and CD163(+) Macrophages Identify Profibrotic Phenotypes in Deceased Hearts With SARS‐CoV‐2 Infections,” Journal of the American Heart Association 12, no. 4 (2023): e027990, 10.1161/JAHA.122.027990. 36789856 PMC10111490

[13] M. Thienel , J. B. Muller‐Reif , Z. Zhang , et al., “Immobility‐Associated Thromboprotection is Conserved Across Mammalian Species From Bear to Human,” Science 380, no. 6641 (2023): 178–187, 10.1126/science.abo5044. 37053338

[14] S. Ito and K. Nagata , “Quality Control of Procollagen in Cells,” Annual Review of Biochemistry 90 (2021): 631–658, 10.1146/annurev-biochem-013118-111603. 33823651

[15] P. S. Bellaye , O. Burgy , P. Bonniaud , and M. Kolb , “HSP47: A Potential Target for Fibrotic Diseases and Implications for Therapy,” Expert Opinion on Therapeutic Targets 25, no. 1 (2021): 49–62, 10.1080/14728222.2021.1861249. 33287600

[16] T. A. Wynn , “Cellular and Molecular Mechanisms of Fibrosis,” Journal of Pathology 214, no. 2 (2008): 199–210, 10.1002/path.2277. 18161745 PMC2693329

[17] G. R. Ross , T. Bajwa, Jr. , S. Edwards , et al., “Enhanced Store‐Operated Ca^2+^ Influx and ORAI1 Expression in Ventricular Fibroblasts from Human Failing Heart,” Biology Open 6, no. 3 (2017): 326–332, 10.1242/bio.022632. 28126709 PMC5374400

[18] G. R. Ross , S. Edwards , C. Warner , et al., “Deletion of Transcription Factor AP‐2α Gene Attenuates Fibroblast Differentiation Into Myofibroblast,” Journal of Cellular and Molecular Medicine 23, no. 9 (2019): 6494–6498, 10.1111/jcmm.14421. 31339227 PMC6714505

[19] V. Nauffal , P. Di Achille , M. D. R. Klarqvist , et al., “Genetics of Myocardial Interstitial Fibrosis in the Human Heart and Association with Disease,” Nature Genetics 55 (2023): 777–786, 10.1038/s41588-023-01371-5. 37081215 PMC11107861

[20] A. J. Wiemer , H. Tong , K. M. Swanson , and R. J. Hohl , “Digeranyl Bisphosphonate Inhibits Geranylgeranyl Pyrophosphate Synthase,” Biochemical and Biophysical Research Communications 353, no. 4 (2007): 921–925, 10.1016/j.bbrc.2006.12.094. 17208200

[21] M. Satoh , K. Hirayoshi , S. Yokota , N. Hosokawa , and K. Nagata , “Intracellular Interaction of Collagen‐Specific Stress Protein HSP47 With Newly Synthesized Procollagen,” Journal of Cell Biology 133, no. 2 (1996): 469–483, 10.1083/jcb.133.2.469. 8609177 PMC2120794

[22] C. C. Farnsworth , M. C. Seabra , L. H. Ericsson , M. H. Gelb , and J. A. Glomset , “Rab Geranylgeranyl Transferase Catalyzes the Geranylgeranylation of Adjacent Cysteines in the Small GTPases Rab1A, Rab3A, and Rab5A,” Proceedings of the National Academy of Sciences of the United States of America 91, no. 25 (1994): 11963–11967, 10.1073/pnas.91.25.11963. 7991565 PMC45356

[23] M. V. Plikus , X. Wang , S. Sinha , et al., “Fibroblasts: Origins, Definitions, and Functions in Health and Disease,” Cell 184, no. 15 (2021): 3852–3872, 10.1016/j.cell.2021.06.024. 34297930 PMC8566693

[24] A. Desmouliere , A. Geinoz , F. Gabbiani , and G. Gabbiani , “Transforming Growth Factor‐Beta 1 Induces Alpha‐Smooth Muscle Actin Expression in Granulation Tissue Myofibroblasts and in Quiescent and Growing Cultured Fibroblasts,” Journal of Cell Biology 122, no. 1 (1993): 103–111, 10.1083/jcb.122.1.103. 8314838 PMC2119614

[25] M. B. Vaughan , E. W. Howard , and J. J. Tomasek , “Transforming Growth Factor‐Beta1 Promotes the Morphological and Functional Differentiation of the Myofibroblast,” Experimental Cell Research 257, no. 1 (2000): 180–189, 10.1006/excr.2000.4869. 10854066

[26] K. A. Lygoe , I. Wall , P. Stephens , and M. P. Lewis , “Role of Vitronectin and Fibronectin Receptors in Oral Mucosal and Dermal Myofibroblast Differentiation,” Biology of the Cell 99, no. 11 (2007): 601–614, 10.1042/BC20070008. 17516912

[27] R. S. Knipe , M. Nurunnabi , C. K. Probst , et al., “Myofibroblast‐Specific Inhibition of the Rho Kinase‐MRTF‐SRF Pathway Using Nanotechnology for the Prevention of Pulmonary Fibrosis,” American Journal of Physiology—Lung Cellular and Molecular Physiology 324, no. 2 (2023): L190–L198, 10.1152/ajplung.00086.2022. 36625494 PMC9925159

[28] Q. Zhang , J. Dong , and Z. Yu , “Pleiotropic Use of Statins as Non‐Lipid‐Lowering Drugs,” International Journal of Biology Sciences 16, no. 14 (2020): 2704–2711, 10.7150/ijbs.42965. PMC7586431 33110390

[29] S. H. Phan , “Biology of Fibroblasts and Myofibroblasts,” Proceedings of the American Thoracic Society 5, no. 3 (2008): 334–337, 10.1513/pats.200708-146DR. 18403329 PMC2645244

[30] S. L. Moores , M. D. Schaber , S. D. Mosser , et al., “Sequence Dependence of Protein Isoprenylation,” Journal of Biological Chemistry 266, no. 22 (1991): 14603–14610. 1860864

[31] S. J. Conway , K. Izuhara , Y. Kudo , et al., “The Role of Periostin in Tissue Remodeling Across Health and Disease,” Cellular and Molecular Life Sciences 71, no. 7 (2014): 1279–1288, 10.1007/s00018-013-1494-y. 24146092 PMC3949008

