# Supplementary figures and images for: Geranylgeranyl Pyrophosphate Promotes Profibrotic Factors and Collagen‐Specific Chaperone HSP47 in Fibroblasts
Source: J Cell Mol Med. 2024 Dec 23;28(24):e70273. doi: 10.1111/jcmm.70273 (PMC11666423; doi:10.1111/jcmm.70273)

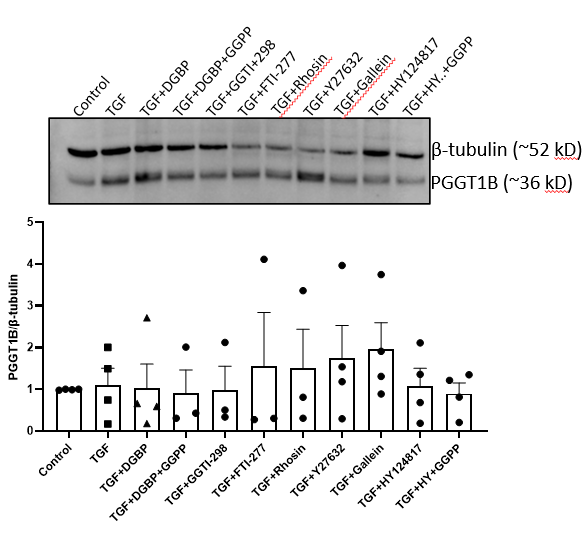
**Supplemental Figure S1**

**Supplemental Figure S2**

**
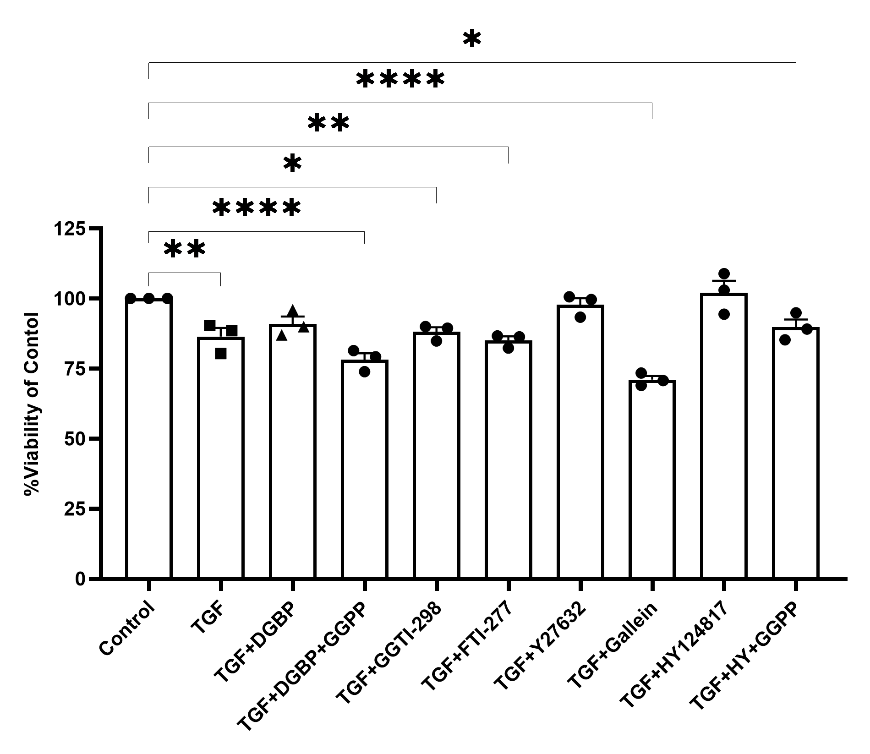
**

Supplement: Supplementary file 1 — Figure S1. Expression of protein geranylgeranyl transferase type I, beta subunit (PGGT1B) during fibroblast/myofibroblast trans‐differentiation. Top panel shows the representative immunoblot of all treatment conditions and bottom panel depicts the bar graph with respective mean values of band intensities. TGF‐β1, increased levels of GGPP, inhibition of GGPS1, inhibition of farnesyl transferase, inhibition of Rho GTPase, inhibition of Rho‐associated kinase (ROCK) or inhibition of G protein βγ subunit‐dependent signalling did not significantly affect the expression of PGGT1B across all treatment conditions. One‐way ANOVA, N = 3; *p < 0.05 considered significant. Figure S2. CellTiter Glo luminescent viability assay. Compared to the control groups, the viability changes across different groups with significance included 86% ± 3% (TGF), 78% ± 2% (TGF + DGBP + GGPP), 88% ± 2% (TGF + GGTI‐298), 85% ± 2% (TGF + FTI‐277), 71% ± 1.3% (TGF + gallein) and 90% ± 3% (TGF + HY124817 + GGPP). However, the overall cellular viability suggests that there was no major cytotoxicity in any group. One‐way ANOVA; N = 3; *p < 0.05 considered significant. [file JCMM-28-e70273-s001.docx]
